# Incidence and Management of Appendiceal Neuroendocrine Tumors in Pediatric Population: A Bicentric Experience with 6285 Appendectomies

**DOI:** 10.3390/children10121899

**Published:** 2023-12-08

**Authors:** Zenon Pogorelić, Vladimir Ercegović, Marko Bašković, Miro Jukić, Ivana Karaman, Ivana Mrklić

**Affiliations:** 1 Department of Pediatric Surgery, University Hospital of Split, 21 000 Split, Croatia; mijukic@kbsplit.hr; 2 Department of Surgery, School of Medicine, University of Split, 21 000 Split, Croatia; vladimir.ercegovic@mefst.hr; 3 Department of Pediatric Surgery, Children’s Hospital Zagreb, 10 000 Zagreb, Croatia; marko.baskovic@kdb.hr; 4 Scientific Centre of Excellence for Reproductive and Regenerative Medicine, School of Medicine, University of Zagreb, Šalata 3, 10 000 Zagreb, Croatia; 5 Department of Pathology, Forensic Medicine and Cytology, University Hospital of Split, 21 000 Split, Croatia; ikaraman@kbsplit.hr (I.K.); ivana.mrklic@mefst.hr (I.M.); 6 Department of Pathology, University of Split School of Medicine, 21 000 Split, Croatia

**Keywords:** neuroendocrine tumor, NET, neuroendocrine neoplasm of appendix, acute appendicitis, appendectomy, appendiceal NET, appendiceal carcinoid tumor, carcinoid

## Abstract

Background: Neuroendocrine tumors (NETs) are rare tumors that arise from neuroendocrine cells and are the most common tumors of the appendix. NETs of the appendix usually cause no symptoms and often go unnoticed until they cause acute appendicitis or are discovered during an accidental appendectomy. As the trend towards the conservative treatment of acute appendicitis increases in the pediatric population, the question arises as to whether the majority of NETs go undetected and are only discovered at an advanced stage. The purpose of the proposed study is to review the incidence and outcomes of treatment for NETs of the appendix in children and include the data presented in the data pool for further review. Methods: From 1 January 2009 to 1 November 2023, a total of 6285 appendectomies were performed in two large pediatric centers in Croatia. After a retrospective review of the case records and histopathologic findings, a total of 31 children (0.49%) were diagnosed with NET of the appendix and included in the further analysis. The primary outcome of this study was the incidence and treatment outcome of pediatric patients diagnosed with NET of the appendix. Secondary outcomes included the patients’ demographic, clinical, and laboratory data and the histopathologic characteristics of tumor species. Results: The overall incidence of NETs of the appendix was stable over the study years, with minor fluctuations. The median age of patients was 14 (interquartile range—IQR: 12, 16) years, with a female predominance (64.5%). The majority of patients (96.8%) presented with acute abdominal pain and underwent appendectomy because acute appendicitis was suspected. Acute appendicitis was confirmed by histopathology in 18 (58%) cases. NETs of the appendix were not detected preoperatively in any of the patients. Among patients with confirmed acute appendicitis, most (*n* = 14; 77.8%) were found to have non-perforated acute appendicitis. In most children, the tumor was located at the tip of the appendix (*n* = 18; 58.1%), and the majority of tumors had a diameter of less than 1 cm (*n* = 21, 67.7%). The mitotic count (*n* = 25, 80.6%) and Ki-67 proliferation index (*n* = 23, 74.2%) were low in most patients, so most tumors were classified as NET G1 (*n* = 25, 80.6%), while NET G2 and NET G3 were found in four (12.9%) and two (6.5%) patients, respectively. All children were treated with appendectomy only. The median follow-up time was 54 (IQR: 24, 95) months. Conclusions: The incidence of appendiceal NET among pediatric patients is very low. NET occurs most frequently in adolescents, with a female predominance. Most tumors are less than 1 cm in diameter, located at the tip, and associated with non-perforated appendicitis. Appendectomy is the treatment of choice, and major surgery was not necessary in our cohort.

## 1. Introduction

Neuroendocrine neoplasms (NENs) of the appendix are rare entities and include neuroendocrine tumors (NETs), neuroendocrine carcinomas (NECs), and mixed neuroendocrine–non-neuroendocrine neoplasms (MiNENs), according to the revised World Health Organization classification (WHO) [1]. Despite their low prevalence, neuroendocrine tumors of the appendix remain the most common tumors of the gastrointestinal tract in children and adolescents [2,3] as well as the most common tumors of the gastrointestinal tract malignancy of the appendix in children [1,4,5,6]. Neuroendocrine tumors of the appendix, formerly called carcinoids or carcinoid tumors, are usually found and diagnosed incidentally after appendectomy due to acute appendicitis upon histopathological examination of the resected appendix [1,5,7,8].

NETs of the appendix are described as slow-growing tumors with excellent prognosis, originating from serotonin-producing enterochromaffin cells [1,8]. According to Duess et al., NETs less than 1 cm in diameter can be treated exclusively by appendectomy, NETs less than 2 cm in diameter most probably have no metastatic potential, and NETs larger than 2 cm in diameter are often treated by right hemicolectomy [8]. The incidence of NETs of the appendix in children ranges from 1 to 1.14 per million per year, but the prevalence of NETs in appendectomies for acute appendicitis ranges from 0.09% to 1.5% [1,9,10]. Preoperative diagnosis of appendiceal NETs is uncommon, which is even more striking in pediatric patients because ultrasound is the most common imaging modality, which can easily miss small lesions [5]. The majority of NETs (80%) arise in the distal portion of the appendix and are smaller than 1 cm in diameter [6].

Acute appendicitis remains one of the most common surgically treated causes of abdominal pain in young adults and children, with an incidence of 1 in 1000 persons per year [11,12]. Although it is a very common pathologic condition in pediatric patients, the exact cause of acute appendicitis remains unknown in some cases. In the remaining cases, luminal obstruction with fecalith or lymphoid hyperplasia is found to be the cause of acute appendicitis. In rare cases, acute appendicitis is caused by neuroendocrine tumors, intestinal parasitic infestations such as *Enterobius vermicularis*, or foreign bodies [13,14].

In our institution and our country, there is a clinical consensus among pediatric surgeons that surgical treatment should be planned in cases of suspected acute appendicitis as well as radiologically proven acute appendicitis [11,12,13,15]. Recently, treatment of acute appendicitis with antibiotics alone has become increasingly popular as an alternative to surgical treatment [16]. As conservative treatment is increasingly replacing surgical treatment, there is a risk of overlooking NETs located in the appendix [5]. There is no consensus on or recommendation regarding the proper further treatment after diagnosing NET of the appendix in children [1,8]. Most children with an NET of the appendix show an uneventful and favorable course of treatment with an excellent prognosis confirmed by postoperative follow-up in the form of somatostatin scans and serum biomarkers [1,6,8].

The purpose of this study is to review the incidence, histopathological characteristics, and outcomes of treatment for neuroendocrine tumors of the appendix in pediatric patients in our cohort and to include the data presented in the data pool for further review.

## 2. Materials and Methods

### 2.1. Patients

**Figure 1:**
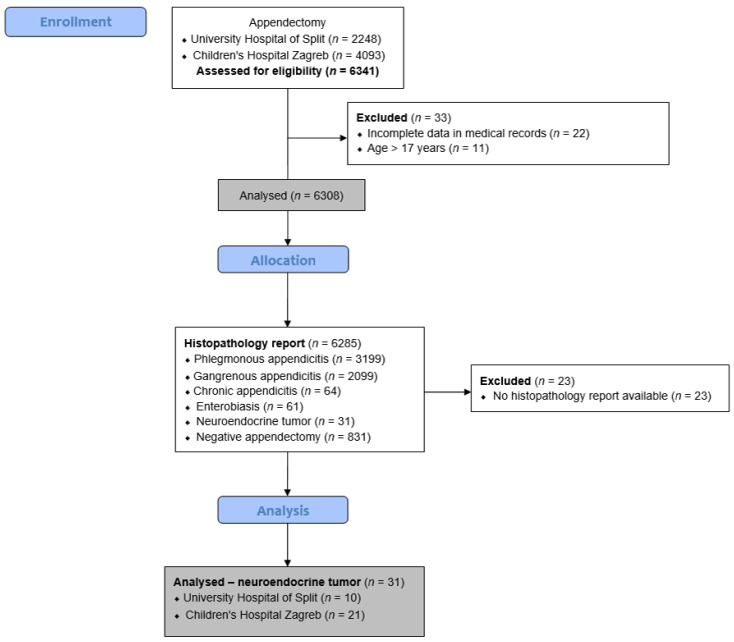
Flowchart of the study.

A retrospective cohort study was performed at two large pediatric centers in Croatia. From 1 January 2009 to 1 November 2023, a total of 6285 appendectomies were analyzed at the Departments of Pediatric Surgery, University Hospital of Split, and Children’s Hospital Zagreb. Out of these, 31 children (0.49%) were diagnosed with an NET of the appendix and included in further analysis. Inclusion criteria were pediatric patients (aged 0 to 17 years) who underwent an appendectomy at either institution for suspected acute appendicitis and were diagnosed with NET of the appendix on histopathology. Patients outside the specified age range, patients without available histopathology reports, NET patients with follow-up less than 6 months, or patients with incomplete data in their medical records were excluded from the study. The flow chart of the study is shown in Figure 1.

### 2.2. Institutional Review Board Statement

The study conformed to the ethical standards of the institutional and national research committee and the 1964 Helsinki Declaration and its later amendments or comparable ethical standards, and the Institutional Review Board of the University Hospital of Split approved the study (approval number: 500-03/23-01/206; date of approval: 26 October 2023).

### 2.3. Outcomes of the Study

The primary outcome of this study was the incidence and treatment outcome of pediatric patients diagnosed with NET of the appendix. Secondary outcomes included patients’ demographic, clinical, and laboratory data and the histopathological characteristics of tumor species.

### 2.4. Data Collection and Study Design

For each patient, basic demographic data (age, sex, weight, height, and body mass index), preoperative laboratory data (white blood cell count, neutrophil granulocytes, and C-reactive protein (CRP)), and clinical findings (abdominal pain localized in the right lower quadrant, rebound tenderness, nausea or vomiting, duration of symptoms, and body temperature) were recorded. In addition, an Appendicitis Inflammation Response (AIR) score was calculated for each patient [11]. Histopathological findings of the vermiform appendix with no inflammatory component were considered negative appendectomies [12]. Findings with described inflammatory components were interpreted as phlegmonous, gangrenous, or chronic inflammation, depending on the inflammatory cells and distribution of the inflammatory component. The diagnosis of phlegmonous or suppurative acute appendicitis was based on transmural neutrophil infiltration in all layers of the appendix [17]. Necrotizing or gangrenous acute appendicitis was diagnosed when transmural inflammation with necrosis was visible microscopically. Chronic appendicitis was diagnosed when predominantly mononuclear inflammatory cells were found in the appendiceal wall and surrounding connective tissue [17,18]. In addition, patients with observed *Enterobiasis* and neuroendocrine tumors of the appendix were considered separate groups. For each patient, the length of hospital stay, reason for appendectomy (elective or urgent appendectomy), type of surgery, complications, surveillance imaging, and follow-up were recorded. Magnetic resonance imaging (MRI), computed tomography (CT), ultrasound (US), and serum 5-hydroxyindoleacetic acid (5-HIAA) were used for the follow-up examinations.

### 2.5. Histopathological Analysis of NETs

**Figure 2:**
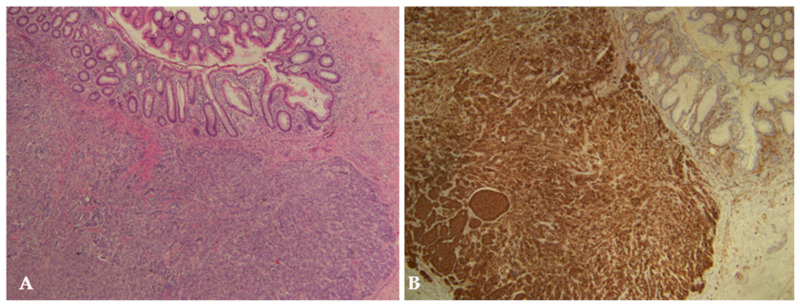
Appendiceal neuroendocrine tumor (NET G1) measuring 9 × 6 mm in a 16-year-old female patient. The tumor is composed of polygonal tumor cells with monomorphic nuclei arranged in nests or trabeculae: (**A**) H&E staining, ×4; (**B**) immunohistochemical staining with CD56, ×4.

The histopathological diagnosis of NET was confirmed by immunohistochemical expression of neuroendocrine markers (mostly synaptophysin and chromogranin) and graded based on the Ki-67 proliferative index and mitotic rate expressed as the number of mitoses per two square millimeters (Figure 2). The following tumor characteristics were recorded for each patient: localization of tumor: apex, middle region, or base of appendix; grade according to the WHO 2019 classification: G1–G3 [1]; size of tumor: <1 cm, 1–2 cm, >2 cm; depth of invasion: invasion of the mesoappendix, vascular and perineural invasion, lymph node invasion; and Ki-67 proliferation index.

### 2.6. Statistical Analysis

Statistical Package for the Social Sciences, SPSS 28.0 (IBM Corp, Armonk, NY, USA), and Microsoft Excel for Windows version 11.0 (Microsoft Corporation, Redmond, WA, USA), were used for statistical analysis. Median and interquartile range (IQR) were used to describe the distribution of quantitative data, whereas categorical data were described with absolute numbers and percentages. Descriptive statistics were used to analyze patients’ demographic, clinical, and laboratory parameters and to determine tumor characteristics and patients’ postoperative outcomes.

## 3. Results

### 3.1. Incidence of NETs of the Appendix

During the study period, a total of 6285 children underwent appendectomy for acute appendicitis. Ultimately, NET of the appendix was diagnosed in 31 (0.49%) patients. The overall incidence of NETs of the appendix was stable during the study years, with minor fluctuations. Acute and chronic appendicitis were histopathologically diagnosed in 5298 (84.29%) and 64 (1.01%) patients, respectively. Enterobiasis was the cause of acute appendicitis in 61 (0.99%) patients. The negative appendectomy rate in the 15-year study period was 831 (13.22%). A summary of histopathological findings is presented in Table 1.

### 3.2. Demographic Characteristics and Clinical Data of Patients with NETs

The demographic characteristics, clinical findings, and laboratory values of patients with NETs of the appendix are shown in Table 2. The median age of patients was 14 (IQR: 12, 16) years, with a female predominance. The median duration of symptoms was 24 h (IQR: 12, 40), and the median body temperature was 37.2 °C (IQR: 36.9, 37.9). Acute abdominal pain and positive rebound/tenderness occurred in almost all patients (96.8%), while nausea and/or vomiting occurred in 74.2% of the cases. The median AIR score was 7 (IQR: 5, 9). In all patients, the diagnosis of acute appendicitis was made preoperatively based on a combination of clinical and laboratory findings along with abdominal ultrasonography. NETs of the appendix were not identified preoperatively in any of the patients by the imaging studies used to diagnose acute appendicitis.

### 3.3. Histopathological Analysis of NETs

A total of 18 patients (58%) with NETs were found to have acute appendicitis on histopathological examination (in 7 cases, phlegmonous appendicitis; and in the remaining 11 cases, gangrenous appendicitis). Other samples were without significant inflammatory changes in the appendix. Among the patients with confirmed acute appendicitis, most (*n* = 14; 77.8%) were found to have non-perforated acute appendicitis. In most of the children, the tumor was located at the tip of the appendix (*n* = 18; 58.1%), and the majority of the tumors were less than 1 cm in diameter (*n* = 21, 67.7%). Mitotic count (*n* = 25, 80.6%) and Ki-67 proliferation index (*n* = 23, 74.2%) were low in most of the patients, so most of the tumors were classified as NET G1 (*n* = 25, 80.6%), while NET G2 and NET G3 were reported in four (12.9%) and two (6.5%) patients, respectively. In nine (29%) patients, the tumor was located in the submucosa; in fifteen (48.4%) patients, the tumor was in the muscularis propria; and in five (16.1%) patients, it was located in the serosa. In two (6.5%) patients, an invasion of the mesoappendix less than 3 cm was found. Perineural invasion was found only in two (6.5%) cases. Furthermore, lymph node invasion was not observed in any of the cases. The detailed histopathological analysis is shown in Table 3.

### 3.4. Outcomes of Treatment

A total of 30 (96.8%) children diagnosed with NET of the appendix underwent emergency surgery for suspected acute appendicitis, except for one case in whom incidental appendectomy was performed. This patient underwent appendectomy during ileocolic resection for Crohn’s disease. All children were treated with appendectomy only. In two cases, re-excision of the mesoappendix remnant was performed because histopathology revealed invasion of the mesoappendix (NET G2 and G3). In both cases, the second histopathological examination was negative for NET. None of the patients underwent ileocolic resection or right hemicolectomy. The median follow-up time was 54 (IQR: 24, 95) months. None of the patients were followed for less than 6 months. During the follow-up period, no patient was found to have a recurrence of NET. None of the patients were lost during the follow-up. The results of treatment are shown in Table 4.

## 4. Discussion

Although NETs are among the most common gastrointestinal malignant tumors in children, they are not sufficiently mentioned in the current scientific literature [19]. The incidence of NET in appendectomies in two large pediatric centers in Croatia was studied over 15 years and revealed an incidence of 0.49%, which is similar to previously published scientific articles [20,21,22,23,24,25]. The average age of our patients at the time of diagnosis was 14 years, with a predominance of females, which is also consistent with previously published data [22,24,26,27]. The current diagnosis of NET is mainly based on clinical symptoms, and NETs are diagnosed after appendectomies by histopathological examination, sometimes even during other surgical procedures such as colectomy, cholecystectomy, and salpingectomy [21,22]. As far as we know, there have been no diagnostic studies focusing only on neuroendocrine neoplasms [28].

The NET in pediatric patients usually occurs at the tip of the appendix, with survival rates as high as 92%, while some studies including only pediatric patients report a 5-year survival rate of nearly 100% [20]. The survival rates of our patients are in line with the current standards. NETs of the appendix metastasize more frequently to the regional lymph nodes than to the liver or other parenchymal organs [21,29,30]. The question has always been how to proceed with treatment after incidental histopathologic identification of appendicular NETs. There are various guidelines for appendectomy or right hemicolectomy, such as the European Neuroendocrine Tumor Society (ENETS) and North American Neuroendocrine Tumor Society (NANETS) guidelines regarding tumor size and mesoappendiceal invasion, while in the pediatric population, the consensus for appendectomy is mostly due to the NET being of lower grade and stage than in adults, leading to more positive outcomes [23,31,32]. All of our patients underwent appendectomy as definitive treatment for their diagnosis, and none of them underwent further resections.

Given the limited amount of published data and the lack of prospective studies, it is of great importance to conduct long-term follow-up studies of selected patients. Sommer et al. pointed out in their study that the following patient groups need special attention and long-term follow-up: patients with NETs more than 2 cm in size, patients with NETs less than 2 cm in size with extension to the mesoappendix, patients with positive surgical margins, and patients with lymph node involvement. A follow-up of at least 10 years is required for the above-mentioned patient groups. If the long-term follow-up of these patients is negative, pediatric patients with NETs of the appendix can be considered disease-free [33].

The indications for major surgery, such as a right colectomy, are controversial. Boxberger et al. examined 237 patients aged up to 19 years and recommended a right hemicolectomy in all patients with NETs of more than 1.5 cm in size due to the significant risk of developing local lymph node metastases. In addition, they recommended ileocecal resection and lymphadenectomy in all patients with NETs of less than 1.5 cm in size with incomplete tumor resection [2]. In contrast to this study, Virgone et al., in their analysis of 113 pediatric patients, advised additional surgery such as ileocecal resection or hemicolectomy only in children with an NET of more than 2 cm in size and at least one positive result on postoperative staging (5-HIAA, US, CT, MRI, or octreotide scintigraphy). In patients with an NET less than 2 cm but with elevated 5-HIAA values and positive octreotide scintigraphy, the authors also recommended additional surgery. They also recommended additional surgery for all cases with positive resection margins [34]. In contrast to previous recommendations, De Lambert et al. found, in their study of 114 patients, that appendectomy alone appears to be curative in all cases, even with incomplete resection, for appendiceal NETs in pediatric patients, with no impact on life expectancy [35].

In the present study, only 2 of 31 patients had an invasion of the mesoappendix. Both underwent additional surgery (re-excision of the mesoappendix remnant). In both cases, the second histopathologic examination was negative for NET. Both patients have been free of recurrence to date, with an average follow-up of 54 months. In their study of 40 patients, Sommer et al. pointed out, and we agree, that invasion of the mesoappendix in pediatric patients should not be considered an indication for additional surgery as it has no impact on treatment outcomes [33]. Njere et al. concluded, in their systematic review of 958 cases, that appendectomy alone is an appropriate treatment for appendicular NET in pediatric patients, regardless of size, location, lymph nodes, or mesenteric involvement. They also pointed out that investigations after the appendectomy proved to be unhelpful [33]. In addition, no deaths and only one recurrence associated with NET of the appendix have been reported in the available literature [23,36].

Nowadays, conservative treatment of acute appendicitis replaces appendectomy in many pediatric centers, and there is a high risk of overlooking NETs of the appendix. As a recently published study shows, there are no reports in the literature of an appendiceal NET with a size of less than 1.5 cm detected by preoperative computed tomography, while some reports confirm that such tumors may be visible on preoperative ultrasound [37]. After successful conservative treatment, interval appendectomy should be considered to detect possible appendiceal NETs, especially if distal lumen dilation is maintained under observation. This statement is strongly supported by the study of 18 patients who were treated conservatively for acute appendicitis and later underwent interval appendectomy; one patient was found to have an appendiceal NET [38].

An analysis of previous scientific publications with larger numbers of cases and long-term follow-up and a review of the literature on NETs of the appendix in children reveals that similar results have been reported in various studies. Many authors agree that appendectomy is the most favorable method in pediatric patients but, at the same time, emphasize the importance of follow-up care [5,23,31,32]. Although our cohort may be limited in terms of sample size, the results of the present study showed similar results in long-term follow-up to some studies with larger samples [33,34,35]. In addition, the potentially subjective measures of pathologic diagnosis of tumor location and size need to be considered. It is known that a certain number of patients who underwent appendectomy have had negative histopathologic findings [39].

The main limitations of the present study are the retrospective design, the relatively limited number of patients, and the follow-up period. More research and clearer diagnostic guidelines can lead to even better treatment outcomes for NETs of the appendix while discouraging more radical surgical approaches that carry a higher risk of morbidity and complications [40]. Collaboration and sharing of clinical data between different centers can provide more accurate percentages for the incidence of NETs of the appendix. Extending follow-up time and adhering to strict guidelines for the treatment of NETs of the appendix can reduce patient mortality to near zero [24,41,42]. Nevertheless, there is still much room for research in the field of neuroendocrine tumors of the appendix, with the most important issue being diagnostics, for which there is still no consensus. Future studies could focus on longer follow-up treatments, the reduction of radical surgical treatments, and the development of precise diagnostic guidelines.

## 5. Conclusions

The incidence of appendiceal NET in pediatric patients is very low. NETs occur most frequently in adolescents, with females outnumbering males. Most tumors are less than 1 cm in diameter, are located at the tip of the appendix, and are associated with non-perforated appendicitis. Performing an appendectomy is an appropriate treatment for NET of the appendix in pediatric patients. The NET of the appendix in pediatric patients has an excellent prognosis.

## Figures and Tables

**Table 1:** Histopathological diagnosis after appendectomy (*n* = 6285).

Histopathological Finding	*n* (%)
Phlegmonous appendicitis	3199 (50.89)
Gangrenous appendicitis	2099 (33.40)
Chronic appendicitis	64 (1.01)
Enterobiasis	61 (0.99)
Neuroendocrine tumor	31 (0.49)
No appendicitis/negative appendectomy	831 (13.22)

**Table 2:** Preoperative characteristics of patients with NETs.

Parameter	Value
Demographic characteristics	
Age (years)	14 (12, 16)
Gender	
Male	11 (35.5)
Female Height (cm) Weight (kg)	20 (64.5) 165 (158, 172) 57 (51, 66)
BMI (kg/m^2^)	21.2 (18.9, 22.2)
Clinical data of patients	
Duration of symptoms (h) Body temperature (°C)	24 (12, 40) 37.2 (36.9, 37.9)
Vomiting/nausea Pain in RLQ Rebound tenderness	23 (74.2) 30 (96.8) 24 (77.4)
AIR score	7 (5, 9)
Laboratory parameters	
Leukocytes (×10^9^/L)	12.9 (9.9, 17.9)
Neutrophils (%)	80 (68, 87)
CRP (mg/L)	35.5 (25.3, 51.1)

Data presented as median (IQR) or *n* (%); BMI—body mass index; RLQ—right lower quadrant; AIR—appendicitis inflammatory response; CRP—C-reactive protein.

**Table 3:** Histopathological characteristics of the NETs.

Variables	*n* (%)
Localization; *n* (%)	
Base of appendix	4 (12.9)
Mid-region of appendix	9 (29)
Tip of appendix	18 (58.1)
Grade; *n* (%)	
NET, G1	25 (80.6)
NET, G2	4 (12.9)
NET, G3	2 (6.5)
Size; *n* (%)	
<1 cm	21 (67.7)
1–2 cm	10 (32.3)
>2 cm	0 (0)
Depth of invasion; *n* (%)	
Submucosa	9 (29)
Muscularis propria	15 (48.4)
Serosa	5 (16.1)
Mesoappendix	2 (6.5)
Invasion of the mesoappendix; *n* (%)	
<3 cm	2 (6.5)
>3 cm	0 (0)
Vascular invasion; *n* (%)	0 (0)
Perineural invasion; *n* (%)	2 (6.5)
Ki-67 proliferation index; *n* (%)	
<3	23 (74.2)
3–20	7 (22.6)
>20	1 (3.2)

NET—neuroendocrine tumor.

**Table 4:** Clinical outcomes of the patients with NETs.

Variables	*n* (%)
Reason for appendectomy	
Acute appendicitis	30 (96.8)
Incidental appendectomy	1 (3.2)
Length of hospital stay; median (IQR)	4 (2, 5)
Complications; *n* (%)	1 (3.2)
Type of surgery; *n* (%)	
Appendectomy	31 (100)
Ileocolic resection/Right hemicolectomy	0 (0)
Surveillance imaging; *n* (%)	
US	21 (67.7)
CT	3 (9.7)
MR	7 (22.6)
Follow up; median (IQR)	
0–1 year	3 (9.7)
1–5 years	13 (41.9)
5–10 years	11 (35.5)
> 10 years	4 (12.9)
Recurrence; *n* (%)	0 (0)

IQR—interquartile range; US—abdominal ultrasound; CT—computed tomography; MR—magnetic resonance.

## Data Availability

The data presented in this study are available upon request to the respective authors. Due to the protection of personal data, the data are not publicly available.
